# Women’s contraceptive discontinuation and switching behavior in urban Senegal, 2010–2015

**DOI:** 10.1186/s12905-018-0529-9

**Published:** 2018-02-05

**Authors:** Janine Barden-O’Fallon, Ilene S. Speizer, Lisa M. Calhoun, Meghan Corroon

**Affiliations:** 1 Maternal & Child Health Department, Gillings School of Global Public Health, University of North Carolina at Chapel Hill, Chapel Hill, USA; 2 Carolina Population Center, University of North Carolina at Chapel Hill, Chapel Hill, USA

**Keywords:** Family planning, Contraception, Discontinuation, Senegal, Urban

## Abstract

**Background:**

With the focus of global and national family planning initiatives on reaching “additional user” targets, it is increasingly important for programs to assess contraceptive method discontinuation and switching. This analysis calculated the discontinuation rate and method-specific discontinuation rates, examined reasons given for contraceptive discontinuation, and assessed characteristics associated with subsequent contraceptive switching and abandonment among women living in urban areas of Senegal.

**Methods:**

Data came from the Measurement, Learning & Evaluation project’s 2015 survey of 6927 women of reproductive age living in six urban sites (Dakar, Pikine, Guédiawaye, Mbao, Kaolack and Mbour). Information on contraceptive use and discontinuation for the five years preceding the survey were recorded in a monthly calendar. Single decrement life tables were used to calculate discontinuation rates. Descriptive analyses were used to assess reasons for discontinuation and method switching after discontinuation. A multinomial logistic regression was used to estimate the likelihood of being a non-user in-need of contraception, a non-user not in-need of contraception, or a method switcher in the month after discontinuation, by sociodemographic and other characteristics.

**Results:**

The 12-month discontinuation rate for all methods was 34.7%. Implants had the lowest one-year discontinuation rates (6.3%) followed by the intrauterine device (IUD) (18.4%) while higher rates were seen for daily pills (38%), injectables (32.7%), and condoms (62.9%). The most common reasons for discontinuation were reduced need (45.6%), method problems (30.1%), and becoming pregnant while using (10.0%). Only 17% of discontinuations were followed by use of another method; most often daily pills (5.2%) or injectables (4.2%). In the multivariate analysis, women with any formal education (primary, secondary or higher) were more than 50% more likely to switch methods than remain in need of contraception after discontinuation than women with no education or Koranic-only education (RRR = 1.59, *p*-value = 0.004; RRR = 1.55, *p*-value = 0.031). The likelihood of switching compared to being “in need” was also significantly higher for women who were married and who discontinued traditional methods.

**Conclusions:**

To support increased contraceptive method use, women with no education and unmarried women are priorities for counseling and information about side effects and method switching at the time of method adoption.

## Background

Family planning (FP) use in West Africa lags behind other regions of the world with an average of 15.3% of married women age 15–49 years using any form of contraception [1]. In recognition of the need to improve FP use across the region, in 2011, the Ouagadougou Partnership created an alliance of Francophone African countries to work together to strengthen programs and accelerate progress towards meeting FP and reproductive health goals [2]. Senegal, a member of the Ouagadougou Partnership, made commitments to the Family Planning 2020 (FP2020) Initiative, thus demonstrating high-level support for FP. These commitments included the launch of a national plan for FP in 2012, increases in the budget allocation for FP activities, the national launch of the Informed Push Model to eliminate stock-outs of contraceptives and supplies, and an investment in demand generation activities nation-wide [3]. As a result, Senegal has made important progress in meeting its FP goals: the 2014 Demographic and Health Survey (DHS) estimated a contraceptive prevalence rate of 22.2%, up from 13.1% in 2010–11; almost a 10-percentage point increase in four years [1].

At the global level, FP2020 aims to reach 120 million additional women and adolescents with FP services by the year 2020 [4]. The nine Ouagadougou Partnership countries aim to contribute at least 2.2 million additional users within the same time frame [2]. The focus on “additional users” rather than “new users” or “first time users” underscores the need for FP programs to ensure that clients have continued access to contraceptive methods and services so that new adopters remain users for as long as they want to delay or avoid pregnancy [5]. The importance of supporting continued use was shown in an analysis of data from 34 countries, which estimated that 38% of women with unmet need (i.e., have a desire to delay or avoid pregnancy but are not using any modern method of family planning) were prior method users who discontinued for one reason or another [6]. Another analysis of data from 36 countries found that about one-third of unintended recent births were attributable to contraceptive discontinuation [7].

Actual discontinuation rates vary by country and by method. A comprehensive analysis of DHS data from 25 countries conducted between 1990 and 2009 found the all-method discontinuation rate to be 38% within the first year of use and 55% by the second year [8]. Earlier work from eight countries found a range of all-method discontinuation rates within the first year of use between 18 and 63% [9]. Discontinuation rates tend to be lower for long-acting methods such as the intrauterine device (IUD) and higher for short-acting methods, such as the pill, injectable, and condoms. For example, first year discontinuation rates were shown to vary from a low of 13% for the IUD to a high of 50% for the condom [8]. These earlier studies did not include implants, a long-term method that is gaining in popularity and has very low discontinuation rates, ranging from 4 to 22% for the first year [10]. Nor have these earlier studies included data from Senegal.

Given that close to half of Senegal’s total population lives in urban areas (44% as of 2017) and that the urban population is growing at 3.6% annually, it is important to consider issues related to contraceptive adoption and continuation in urban populations in the country [11]. Further, while there is generally greater access to health facilities in cities than in rural areas, this urban advantage may not be found among all residents [12, 13]. In particular, urban poor women may lack access to information and services and may have discontinuation rates that are higher than their wealthier counterparts. This study used data from a large urban sample in Senegal to examine women’s discontinuation rates and patterns and the demographic factors associated with method switching. The specific objectives of this analysis were to calculate overall and method-specific discontinuation rates, examine reasons given for discontinuation, and assess characteristics associated with subsequent contraceptive switching and abandonment among women living in six urban areas of Senegal.

## Methods

The data for this analysis were collected by the Measurement, Learning & Evaluation (MLE) project as part of the evaluation of the Urban Reproductive Health Initiative in Senegal, a Bill & Melinda Gates Foundation-funded initiative to increase the use of modern contraceptive methods, especially in disadvantaged urban populations. Data were collected by face-to-face interview from 6927 women in 2015 as the third round of a panel study (73.5% of the 2011 baseline sample were found and surveyed in 2015). Women were aged 15–49 years at baseline and living in the urban areas of Dakar, Pikine, Guédiawaye, Mbao, Kaolack and Mbour. These cities were selected for intervention by the Senegal Urban Reproductive Health Initiative, called the Initiative Sénégalaise de Santé Urbaine, and may not represent urban Senegal overall. Half of the sampling units were located in poor areas and half were located in non-poor areas. The definition of poor/non-poor was based on a participatory rating process conducted with municipal level administrative staff and other leaders. Questions were asked across the five dimensions of the UNHABITAT definition of slums [14]. All study procedures were approved by the Comité National d’Ethique pour la Recherche en Santé through the Ministry of Health in Senegal and by the Institutional Review Board of at the University of North Carolina at Chapel Hill.

Information on contraceptive use and discontinuation for the five years preceding the 2015 survey were recorded in a monthly calendar, similar to what is used in the DHS. The calendar captured month-by-month data on contraceptive use, non-use, source of method, discontinuation, reason for discontinuation, pregnancy, birth, pregnancy terminations, and whether married/living with a man. Contraceptive methods recorded in the calendar included sterilization, implant, IUD, injectable, daily pill, condom, lactational amenorrhea method (LAM), other modern (Standard Days Method, emergency pill, and “other modern” as reported by women) and traditional methods. Source of contraceptive method (coded as pharmacy, private facility, public facility, or other/don’t know/missing) was assessed for each contraceptive event. Marital union (coded as in union vs. not in union) was assessed at the end of each event. The survey questionnaire collected information on socio-demographic characteristics, such as age, education, parity, city of residence, and household wealth. For this analysis, women’s characteristics were coded as categorical variables: age (18–24, 25–29, 30–34, 35–39, or 40–54); education (none/Koranic-only/missing, primary, or secondary or higher); parity (0, 1–2, 3–4, 5+); city (Dakar, Pikine, Guédiawaye, Mbao, Kaolack or Mbour); household wealth (lower two quintiles, middle quintile, or higher two quintiles). Wealth was calculated using a similar method as DHS using principal components methods [15].

Single decrement life tables were used to calculate all-reason and method-specific discontinuation rates at 6, 12, 18, and 24 months among women who started an episode of use during the calendar period. An “episode of use” was defined as a continuous period of contraceptive use (conversely, an “episode of non-use” was a continuous period of no contraceptive use). An “event”, which can start or stop an episode of use or non-use, was defined as any status captured by the calendar including contraceptive use, non-use, pregnancy, birth, or pregnancy termination. An event could be as short as one month or as long as the whole calendar period. No censor period was used.

Further descriptive analyses were conducted for women who discontinued use of a method at least once during the calendar period. For these women, we examined reasons for discontinuation and method switching in the month after discontinuation. Reason for discontinuation was categorized as reduced need (i.e., wanted to become pregnant; difficult to get pregnant/menopausal; infrequent sex/husband away; marital dissolution/separation), method problem (i.e. wanted more effective method; fear of side effects/health concerns; inconvenient to use; created menstrual problems; gained weight; did not like method; lack of sexual satisfaction; lack of privacy for use), method failure/became pregnant while using, husband disapproved, supply (lack of access/too far, costs too much), and “Other”/missing/fatalistic/Don’t know.

A “Status of next event” variable was constructed based on the calendar month following the discontinuation and was coded as “No need” when no method was started and the reason for discontinuation was wanting to become pregnant, actual pregnancy, menopause, infrequent sex, or marital dissolution (i.e. reduced need). “In need” was the classification when no method was started and any other reason for discontinuation was given. “Method switch” was the classification for discontinuations followed by the start of any method in the month following the discontinuation. A multinomial logistic regression was then used to estimate the likelihood of in need, no need, or method switch status after discontinuation, by women’s socio-demographic characteristics, the method discontinued, and the source of method discontinued. Methods discontinued were categorized as daily pill, injectable, implant/IUD, other modern, or traditional, for the regression analysis. Relative risk ratios were interpreted for the predictor variables, with statistical significance indicated at *p* < 0.05. Regression analyses were adjusted for clustering (women can contribute multiple discontinuations). Analyses were run using STATA 14.1. The descriptive analyses were weighted for survey design; reported percentages reflect the weighting. Discontinuation rates and number of observations are presented as unweighted.

## Results

Characteristics of the women included in the survey are shown in Table 1. Women included in the 2015 MLE endline longitudinal survey were generally older than a representative urban sample due to the longitudinal design. More than half of the women were age 30 or older, while only 3.9% were in the youngest age category. Most women had received some education, though only 37.2% had attained some secondary or higher level. The number of children ever born ranged from 0 to 14. A solid proportion of the sample, 34.9%, had not had any children. The highest percentage of women in the sample lived in Dakar (41.4%). Residency in Mbao was also common (22.7%). The other four cities together accounted for 35.9% of women.

**Table 1:** Percent distribution of socio-demographic characteristics of women and calendar events, Senegal, 2015^a^

Socio-demographic characteristics	%
Age	
15–19	3.9
20–24	22.1
25–29	21.3
30–34	16.2
35–39	13.5
40–54	23.0
Education	
None/Koranic-only/missing	31.3
Primary	31.5
Secondary or higher	37.2
Parity	
Zero	34.9
One-two	26.2
Three-four	21.1
Five+	17.7
City	
Dakar	41.4
Pikine	11.0
Guédiawaye	10.7
Mbao	22.7
Kaolack	7.5
Mbour	6.8
Wealth	
Lower	42.2
Middle	19.7
Higher	38.2
Cluster-level	
Poor	48.0
Non-poor	52.0
Calendar Events	%
Number of births per woman	
Zero	57.4
One	29.1
Two	12.0
Three	1.4
Four	0.0
Number of abortions, miscarriages, stillbirths per woman	
Zero	92.8
One	6.4
Two	0.7
Three	0.1
Number of discontinuations per woman	
Zero	72.2
One	19.2
Two	6.8
Three	1.5
Four	0.3
Five+	0.0
Total	100.0

*N* = 6927

^a^Percentages are weighted for survey design

Of the 6927 women included in the survey, 60.8% did not use any contraception during the calendar period, 11.4% used contraception and did not have any discontinuations and 27.8% used contraception and had at least one discontinuation (results on events not shown). The five-year calendar captured a total of 26,022 events for these women. These events included 5153 pregnancies; 598 abortions, miscarriages, and stillbirths; 4334 births; 4511 contraceptive episodes (i.e. periods of continuous use); and 11,436 episodes of non-use (i.e. period of continuous non-use). Women were married or in union at the end of 89.8% of events. Of the 4511 contraceptive episodes, the most common methods used were injectables (32.3%), daily pill (28.5%), implants (12.8%) and condoms (8.0%). There was a total of 2678 discontinuations. The number of events per woman are shown in Table 1 under “Calendar Events”. Almost 43% of women had at least one birth during the period of the calendar; 13.5% had two, three or four births. 7.2% of women experienced at least one pregnancy termination. The number of discontinuations per woman ranged between 0 and 8. Overall the mean number of discontinuations per woman was 0.4; among women who discontinued at least once during the calendar period, the mean number of discontinuations was 1.4.

The all-method and method-specific discontinuation rate at 6, 12, 18, and 24 months is shown in Table 2. The 12-month discontinuation rate for all methods was 34.7% and increased to 53.7% by 24 months. Implants had the lowest one-year discontinuation rates (6.3%) followed by the IUD (18.4%). By definition, all LAM users discontinued by 12 months since LAM is only a method for the first six months postpartum. “Other modern” methods, though contributing a very small number of episodes, also had a very high rate of discontinuation (93%). This is likely due to the emergency pill, which is not typically viewed as a continuous-use method and thus only reported in the month of use. Of the commonly used short-term methods, the 12-month discontinuation rate for daily pills was 38%, for injectables was 32.7%, and for condoms was 62.9%.

**Table 2:** Discontinuation rates at 6, 12, 18, and 24 months, by method (all reasons), Senegal 2015

Method	6 months	12 months	18 months	24 months	No. of episodes of use
Sterilization	*NA*				14
Implant	3.3	6.3	10.0	15.0	569
IUD	13.5	18.4	20.5	26.8	155
Injectables	15.3	32.7	44.9	56.5	1260
Daily pill	25.5	38.0	52.4	59.8	1027
Condoms	51.5	62.9	72.0	80.7	203
LAM	13.3	100.0	100.0	100.0	116
Other modern^a^	81.2	93.0	93.0	100.0	48
Traditional	25.9	40.7	52.2	62.9	242
All methods	19.7	34.7	45.2	53.7	3634

Among women age 15–49 at baseline who started an episode of contraceptive use during the calendar period, *N* = 3634

^a^Includes Standard Days Method, emergency pill, and “other modern” as reported by women

Table 3 presents the reasons for discontinuation by method. Of the 2678 discontinuations, the most common reasons given by women included reduced need (i.e., wanted to become pregnant; difficult to get pregnant/menopausal; infrequent sex/husband away; marital dissolution/separation) (45.6%), method problems (i.e. wanted more effective method; fear of side effects/health concerns; inconvenient to use; created menstrual problem; gained weight; did not like method; lack of sexual satisfaction; lack of privacy for use) (30.1%), and method failure/became pregnant while using (10.0%). A total of 14.4% of discontinuations were due to husband disapproval (2.7%), “other” (9.4%), missing/don’t know (1.8%), or supply (0.5%). Unlike the other contraceptive methods shown, discontinuations of the IUD were more likely to be due to a method problem (53.6%) than any other reason. Method failures were most likely to occur with traditional methods (18.8%), condoms (16%) or the daily pill (13.7%). Husband disapproval was given as a reason for discontinuation most often with condoms (5.6%).

**Table 3:** Percent distribution of main reason for discontinuation, by method, Senegal 2015

Reason	Implant	IUD	Injectable	Daily pill	Condoms	LAM	Other modern	Traditional	All
Reduced need	64.5	26.9	43.9	49.1	57.8	2.1	25.4	42.5	45.6
Method problem^a^	26.8	53.6	41.9	24.8	12.4	25.6	13.5	26.8	30.1
Method failure/became pregnant while using	0.0	4.3	5.2	13.7	16.0	0.0	6.7	18.8	10.0
Husband disapproved	0.8	0.7	1.4	3.8	5.6	0.0	0.0	2.6	2.7
Supply (Lack of access/too far, costs too much)	0.0	0.0	1.2	0.3	0.1	0.0	0.0	0.0	0.5
“Other”	7.4	9.3	5.6	6.3	5.3	70.9	52.7	5.6	9.4
Missing/DK	0.6	3.6	0.9	2.1	2.7	1.3	1.5	3.8	1.8
Total	100	100	100	100	100	100	100	100	100
Number of discontinuations	143	66	985	951	176	114	45	198	2678

*N* = 2678; percentages are weighted for survey design

^a^Wanted more effective method; fear of side effects/health concerns; inconvenient to use; created menstrual problem; gained weight; did not like method; lack of sexual satisfaction; lack of privacy for use

As shown in Table 4, most discontinuations were not followed by a method switch in the month following the discontinuation: 69.4% were followed by non-use and 13.3% were followed by a pregnancy. Only 17% of discontinuations were followed by use of another method. In general, daily pills were the most common method used after discontinuation (5.2%), followed by injectables (4.2%). Either the implant or IUD were used after 3.2% of discontinuations. When the next event was method use, discontinuations of long acting methods were most likely followed by use of short term methods such as injectables (6.1% after implant discontinuation and 8.0% after IUD discontinuation) and daily pills (5.6% after implant discontinuation and 8.4% after IUD discontinuation). Pregnancy was highest after discontinuation of traditional methods (20.8%) and the daily pill (19.7%). The next event after discontinuation was also assessed for the sub-group of discontinuations that were not due to reduced need (*n* = 1514). A similar pattern to that presented in Table 4 was found, though switching was more common (32%) and pregnancy was highest after discontinuation of condoms (38.8%) and traditional methods (30.8) (contact first author for results).

**Table 4:** Percent distribution of next event after discontinuation, by method discontinued, Senegal 2015^a, c^

Method Discontinued	Next event after discontinuation										
	No method	Implant	IUD	Injectables	Daily pill	Condoms	Other modern	Trad method	Pregnancy	Termination/Stillbirth	Total n %
Implant	84.0	0.0	0.6	6.1	5.6	0.6	0.0	2.2	0.9	0.0	143,100%
IUD	69.8	5.7	0.0	8.0	8.4	0.0	0.0	0.0	8.1	0.0	66,100%
Injectables	72.4	2.8	0.6	0.0	11.5	1.9	0.0	3.2	7.6	0.0	985,100%
Daily pill	66.8	2.0	1.9	7.1	0.0	0.3	0.3	1.6	19.7	0.4	950,100%
Condoms	71.5	2.1	0.2	3.9	2.1	0.0	1.0	2.1	17.0	0.0	173,100%
LAM	72.0	3.2	0.0	3.0	3.5	0.5	0.0	17.5	0.2	0.0	114,100%
Other modern^b^	74.1	0.0	0.0	0.2	9.6	5.4	0.0	2.8	7.9	0.0	44,100%
Traditional	55.8	1.7	0.0	7.2	3.7	4.0	1.4	3.7	20.8	1.7	198,100%
Total %	69.4	2.3	0.9	4.2	5.2	1.3	0.3	3.0	13.3	0.3	2,673^b^ 100%

*N* = 2673

^a^Percentages are weighted for survey design

^b^Includes Standard Days Method, emergency pill, and “other modern” as reported by women

^c^Missing (*n* = 5)

In total, 512 (17%) discontinuations were followed by a method switch; 1452 (56.2%) discontinuations were followed by non-use and were also classified as “no need” for contraception, based on the reason given for the discontinuation. Finally, 709 discontinuations (26.8%) were followed by non-use, and were classified as “in need” of contraception, based on the reason given for discontinuation.

Results from a multinomial logistic regression of the relative risk of being “in need” relative to “no need”, and “in need” relative to “method switch”, illuminated several interesting patterns. As seen in Table 5, the relative risk of discontinuation for married women was 75% more likely to result in a status of “no need” for contraception versus “in need” compared to unmarried women. Being age 35–39 years had a similar effect, whereas parity of one or more and residence in Mbour urban area were associated with a lower likelihood of being “no need” versus “in need”. Compared to the daily pill, discontinuations of injectables, implants, or other modern methods were less likely to result in “no need” than “in need”. Likewise, compared to pharmacies as a method source for the discontinued method, discontinuations of methods obtained from public, private, or other sources are less likely to result in “no need” compared to “in need”.

**Table 5:** Relative risk of being in need, not in need, or method switcher after discontinuation, Senegal 2015

	Status after discontinuation [Comparison is “In Need”]			
	No Need		Method Switch	
	RRR	P > |z|	RRR	P > |z|
Age				
15–24	Reference		Reference	
25–29	1.14	0.513	1.08	0.774
30–34	1.46	0.061	0.83	0.474
35–39	1.66	0.018*	1.25	0.426
40–54	0.99	0.965	0.92	0.770
Education				
None/Koranic-only	Reference		Reference	
Primary	1.20	0.130	1.59	0.004*
Secondary or higher	0.99	0.929	1.55	0.031*
Parity				
0	Reference		Reference	
1–2	0.36	0.018*	1.96	0.539
3–4	0.30	0.008*	1.27	0.339
5+	0.22	0.001*	1.22	0.316
City				
Dakar	Reference		Reference	
Guediawaye	1.23	0.307	1.32	0.272
Pikine	1.15	0.515	0.93	0.801
Mbao	0.73	0.099	0.50	0.009*
Mbour	0.62	0.003*	0.87	0.513
Kaolack	1.21	0.272	0.65	0.082
Wealth				
Lower 40%	Reference		Reference	
Middle 20%	1.06	0.674	1.13	0.519
Upper 40%	1.09	0.498	1.00	0.990
Marital status				
Married/In-union	1.75	0.012*	2.08	0.050*
Not in union	Reference		Reference	
Method discontinued				
Daily Pill	Reference		Reference	
Injectables	0.55	0.000*	1.08	0.580
Implant/IUD	0.45	0.000*	0.76	0.293
Other modern	0.17	0.000*	1.01	0.977
Traditional	1.77	0.079	2.93	0.005*
Source of method				
Pharmacy	Reference		Reference	
Public	0.36	0.000*	0.70	0.327
Private	0.39	0.002*	0.59	0.102
Other/DK/Missing	0.36	0.000*	0.69	0.263
Constant	12.96	0.000	0.30	0.090

*N* = 2673

Standard errors are adjusted for 1923 clusters (individual women) in the data

*p < 0.05

Fewer variables showed statistical significance in the comparison of method switching after discontinuation to being “in need”. The relative risk for switching methods was significantly lower for women living in Mbao. However, women with any formal education (primary, secondary or higher) were more than 50% more likely to switch methods than remain in need of contraception after discontinuation than women with no education or Koranic-only education. The likelihood of switching compared to being “in need” was also significantly higher for women who were married and for women who had discontinued traditional methods compared to those who discontinued the daily pill.

## Discussion

In line with previously cited literature, the first-year all-method discontinuation rate reported by the recent Senegal DHS was 35%, though there was variation by method; 46% for daily pills, 41% for injectables, and 8% for implants [16]. The most common reasons for discontinuation were wanting to become pregnant (33%), health problems or side effects from using the method (23%), and infrequent sex/husband is absent (12%) [16]. Contraceptive discontinuation in the sample of women living in urban Senegal was similar to that found for the country as a whole, though method-specific discontinuation of the most commonly used methods was found to be slightly lower in our urban sample. The top reasons for contraceptive discontinuation - reduced need (mainly the desire to become pregnant) and method problems (mainly the fear of side effects and health concerns) were also similar to what was reported in the Senegal DHS [16].

Large differences are known to exist across countries in the rate of method switching after discontinuation. An examination of DHS data from 23 countries found that within three months after method discontinuation, 64% of women in Morocco switched to a modern method while only 17% of women in Malawi had done so [17]. Our analysis found that like Malawi, method switching was relatively uncommon in urban Senegal- only 17% of discontinuations were followed by use of a modern method in the next month. Discontinuation has previously been shown to account for more than one third of unmet need [6]. Our assessment of the reasons for discontinuation and contraceptive use status in the month following discontinuation found that about 27% of discontinuations resulted in women who were “in need” or had an unmet need for contraception. The findings of the multivariate analysis indicated that women were most likely to have an unmet need for contraception after discontinuation (i.e., discontinue in need) if they were unmarried, had no education or Koranic-only education, lived in the urban areas of Mbour or Mbao, had at least one child, discontinued a modern method other than the pill (daily pill discontinuers were the most likely to switch methods), or obtained contraceptive methods at sources other than pharmacies. Women with these characteristics are priorities for counseling and provision of information about side effects and method switching at the time of method adoption. Similar to the well-documented positive relationship of women’s education on use of family planning, this analysis found that women’s education had a significant association with method switching as well. Economic status was not found to be significantly related to having unmet need after discontinuation. The IUD had a high rate of discontinuation due to method-related problems, specifically menstrual problems and the fear of side effects and health concerns; women adopting this method need to receive full counseling on expectations and potential side effects associated with use of the method. We found that women who were users of traditional methods were significantly more likely to be switchers to modern methods; traditional method use may be a gateway to more effective method use and therefore these women should be supported in this process. A recent analysis of data collected at health facilities in Senegal found that the general level of counseling for FP was inadequate: only 18% of providers counseled their clients on three items examined by the survey (how to correctly use the method, possible side effects, and when to make a return visit) [18]. Yet earlier research in Senegal found that women who received good quality care at the time of method initiation were 1.3 times more likely to be using a method after 16 months than were women who did not receive higher quality counseling [19]. Such findings indicate that much work needs to be done to improve FP use in Senegal.

This analysis benefited from data collected from a unique sample of women in six urban areas of Senegal. The data collected from a contraceptive calendar allowed for detailed assessment of contraceptive discontinuation and switching patterns in urban areas, beyond what is available in the national-level DHS. The analysis was limited by recall bias in retrospective calendar data; this could include inaccuracies in reporting contraceptive histories as well as reclassification of reasons for discontinuation, particularly when it was due to method failure. The results presented in the paper may undercount discontinuation due to method failure, as the three months prior to the date of the interview were included in the analysis. As a result, the numbers for pregnancy and pregnancy due to method failure may be underreported. The trade-off was an increase in the amount of data, including discontinuations, to include in the analysis, which is an important consideration as the use of contraception, and thus the number of discontinuations, were relatively low in Senegal. Furthermore, some prior studies accepted a period of three months after discontinuation in which method switching can occur (for example, in Ali, Cleland, and Shah, 2012) [8]. Our analysis limits the period for switching to one month following discontinuation due to the importance of maintaining protection against unintended pregnancy. Maintaining contraceptive coverage and enabling women to switch methods immediately is one of the key service delivery interventions identified in a recent review of literature on contraceptive discontinuation [20]. Our data would include an additional 56 method switches with an extended three-month period; we did not run separate analyses with these additional switches.

## Conclusions

While there is no “optimal” level of method continuation for FP methods, it is nonetheless important for programs to monitor and evaluate method discontinuation, especially when considered alongside method switching to better understand use trends in a given context or population. The results of our analysis suggest that method discontinuation is associated with Senegal’s low CPR. Additionally, immediate method switching to other modern FP methods is low and can be improved. This type of information on contraceptive use patterns can benefit FP programming and implementation and bring attention to issues related to discontinuation, specifically discontinuation while still in need, and method switching. Issues of programmatic interest include maintaining an optimal method mix at facilities, which may include increasing the number of methods available, and ensuring there is a mix of methods at all distribution sources. Programs should provide comprehensive counseling and education on side effects and health concerns, dispelling myths and rumors about contraception, and how to safely and effectively switch methods when needed. Assisting women to maintain contraceptive coverage after method discontinuation will help ensure that Senegal is meeting the FP needs of its population and contribute to the achievement of its national FP goals.

## Abbreviations

DHS: Demographic and health survey
FP: Family planning
FP2020: Family Planning 2020
IUD: Intrauterine device
LAM: Lactational amenorrhea method
MLE: Measurement, Learning & Evaluation
